# Supplementary material for: Structure of the human KMN complex and implications for regulation of its assembly
Source: Nat Struct Mol Biol. 2024 Mar 8;31(6):861–73. doi: 10.1038/s41594-024-01230-9 (PMC11189300; doi:10.1038/s41594-024-01230-9)
Supplement: Supplementary file 1 — Supplementary Figs. 1 and 2. [file 41594_2024_1230_MOESM1_ESM.pdf]

nature structural & molecular biology

Article

https://doi.org/10.1038/s41594-024-01230-9

Structure of the human KMN complex and implications for regulation of its assembly

In the format provided by the authors and unedited

Structure of the human KMN complex and implications for regulation of its assembly

By Soumitra Polley, Tobias Raisch *et al.*

Supplementary Information

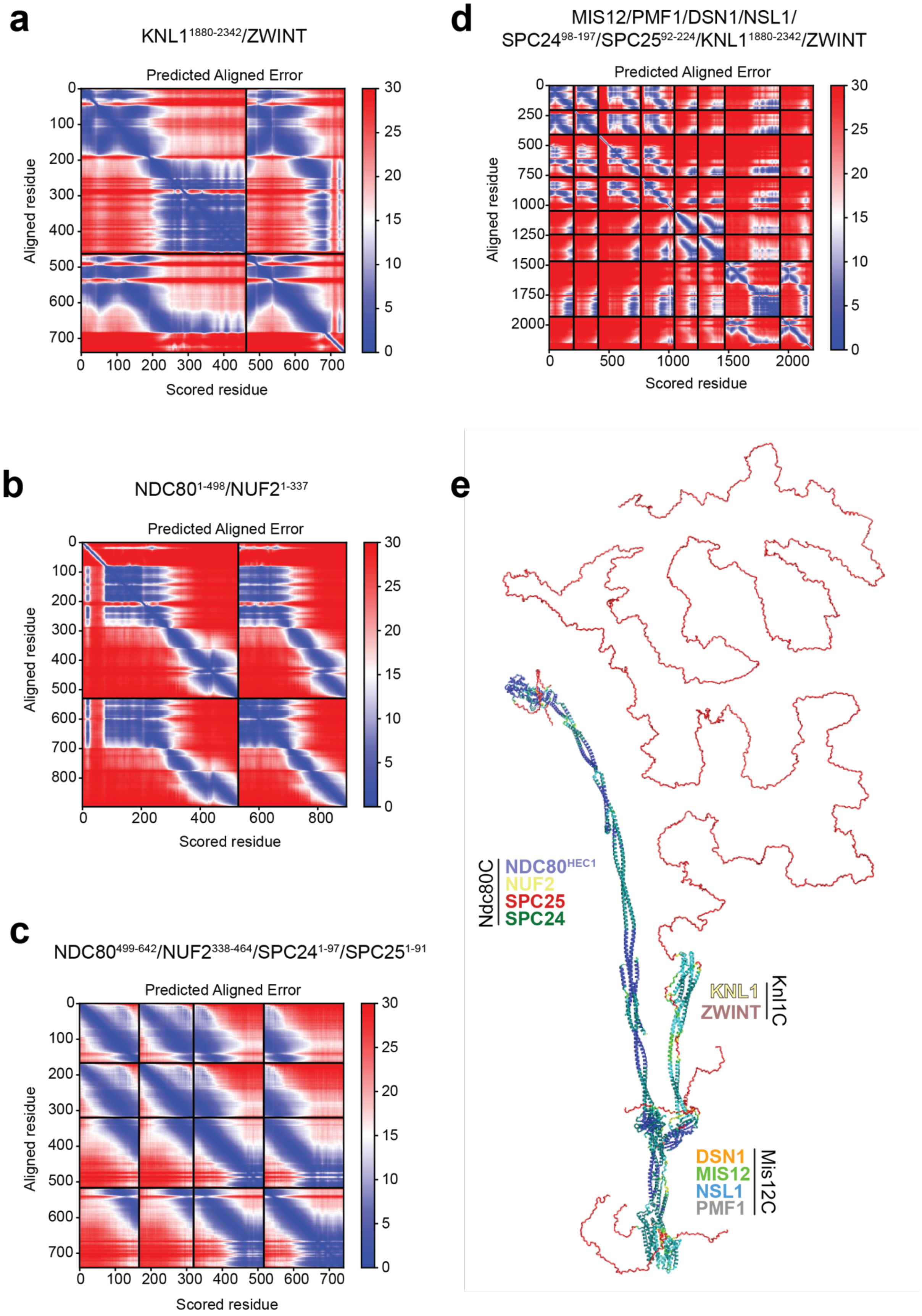
a
KNL1$^{1880-2342}$/ZWINT
Predicted Aligned Error
Aligned residue
Scored residue
d
MIS12/PMF1/DSN1/NSL1/
SPC24$^{98-197}$/SPC25$^{92-224}$/KNL1$^{1880-2342}$/ZWINT
Predicted Aligned Error
Aligned residue
Scored residue
b
NDC80$^{1-498}$/NUF2$^{1-337}$
Predicted Aligned Error
Aligned residue
Scored residue
e
Ndc80C
NDC80^{HEC1}
NUF2
SPC25
SPC24
KNL1
ZWINT
Knl1C
DSN1
MIS12
NSL1
PMF1
Mis12C
c
NDC80$^{499-642}$/NUF2$^{338-464}$/SPC24$^{1-97}$/SPC25$^{1-91}$
Predicted Aligned Error
Aligned residue
Scored residue

Supplementary Figure 1 ***AF2 predictions***

(**a**-**d**) AF2 predicted aligned error (PAE) plots for predictions of the indicated constructs in the indicated succession (each chain is delimited by a / sign) and with the indicated residue boundaries. The human $KNL1_{1880-2342}$/ZWINT prediction is depicted in Figure 6a. (**e**) Per-residue confidence scores (pLDDT) mapped on the model displayed in Figure 7. Dark blue indicates highest confidence, red lowest confidence/disordered segments.

*adjusted intensity

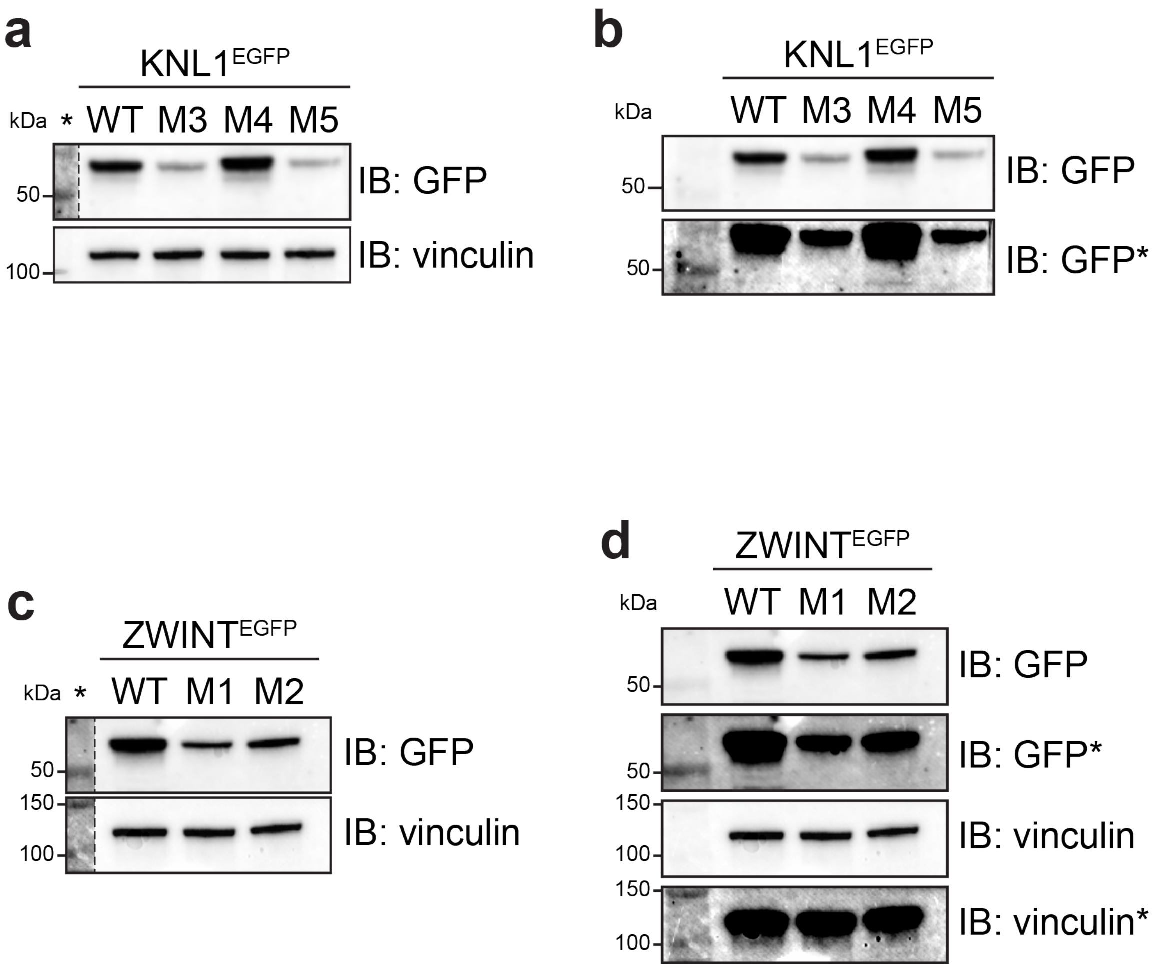

Supplementary Figure 2

Supplementary Figure 2 ***Western blots at normal and adjusted intensities***

(**a**,**c**) Western blots with markers as shown in Extended Data Figure 4g and Extended Data Figure 10h, respectively. (b,d) Comparison of Western blots shown at normal or adjusted intensity (the latter indicated by an asterisk). To demonstrate the position of the molecular weight markers, we merged the marker from the adjusted intensity image with the normal intensity image, as in the latter the signal for the marker was very weak. The vertical line in panels a,c signals the merger from the same blot displayed at different intensities.
