# Supplementary material for: Structure of the human KMN complex and implications for regulation of its assembly
Source: Nat Struct Mol Biol. 2024 Mar 8;31(6):861–73. doi: 10.1038/s41594-024-01230-9 (PMC11189300; doi:10.1038/s41594-024-01230-9)
Supplement: Supplementary file 2 — Reporting Summary [file 41594_2024_1230_MOESM2_ESM.pdf]

nature portfolio

Corresponding author(s): Andrea Musacchio

Last updated by author(s): Jan 5, 2024

Reporting Summary

Nature Portfolio wishes to improve the reproducibility of the work that we publish. This form provides structure for consistency and transparency in reporting. For further information on Nature Portfolio policies, see our Editorial Policies and the Editorial Policy Checklist.

Statistics

For all statistical analyses, confirm that the following items are present in the figure legend, table legend, main text, or Methods section.

n/a	Confirmed	
☐	☒	The exact sample size (*n*) for each experimental group/condition, given as a discrete number and unit of measurement
☐	☒	A statement on whether measurements were taken from distinct samples or whether the same sample was measured repeatedly
☐	☒	The statistical test(s) used AND whether they are one- or two-sided<br>*Only common tests should be described solely by name; describe more complex techniques in the Methods section.*
☒	☐	A description of all covariates tested
☒	☐	A description of any assumptions or corrections, such as tests of normality and adjustment for multiple comparisons
☐	☒	A full description of the statistical parameters including central tendency (e.g. means) or other basic estimates (e.g. regression coefficient) AND variation (e.g. standard deviation) or associated estimates of uncertainty (e.g. confidence intervals)
☒	☐	For null hypothesis testing, the test statistic (e.g. *F*, *t*, *r*) with confidence intervals, effect sizes, degrees of freedom and *P* value noted<br>*Give P values as exact values whenever suitable.*
☒	☐	For Bayesian analysis, information on the choice of priors and Markov chain Monte Carlo settings
☒	☐	For hierarchical and complex designs, identification of the appropriate level for tests and full reporting of outcomes
☒	☐	Estimates of effect sizes (e.g. Cohen's *d*, Pearson's *r*), indicating how they were calculated

Our web collection on statistics for biologists contains articles on many of the points above.

Software and code

Policy information about availability of computer code

Data collection

EPU v2.7 Thermo Fisher Scientific
Image Lab Bio-rad https://www.bio-rad.com/de-de/product/image-lab-software?ID=KRE6P5E8Z
Slidebook, 3i (Intelligent Imaging Innovations)
FortéBio Octet BLI Data acquisition software
Deltavision SoftWoRx software

Data analysis

cryoSPARC Version 4.2.1 (Punjani et al. 2017, https://cryosparc.com/)
TranSHIRE v1.4 (Stabrin et al., 2020 https://transphire.readthedocs.io/en/latest/
MOTIONCOR2 v1.3.0 Zheng et al., 2017 http://msg.ucsf.edu/em/software/motioncor2.html
CTFFIND4 (Rohou and Grigorieff, 2015 http://grigoriefflab.janelia.org/ctffind4)
MOTIONCOR2 (Zheng et al., 2017 http://msg.ucsf.edu/em/software/motioncor2.html)
SPHIRE v1.5 (Moriya et al., 2017 http://sphire.mpg.de)
crYOLO v1.7 (Wagner et al., 2019 https://cryolo.readthedocs.io/en/stable/)
RELION v3.1.2 (Scheres Lab https://www3.mrc-lmb.cam.ac.uk/relion/index.php?title=Main_Page)
Pymol v2.5.4 Schrödinger, LLC https://pymol.org/2/
COOT v0.9.8.2 (Emsley et al., 2010 https://www2.mrc-lmb.cam.ac.uk/personal/pemsley/coot/)
PHENIX v1.20.1 (Adams et al., 2010 https://www.phenix-online.org)
DeepEMhancer v0.14 (Sanchez-Garcia et al., 2021 https://github.com/rsanchezgarc/deepEMhancer)
AlphaFold-Multimer v3.2.1 Evans et al., 2021
DynDom6D (Veevers and Hayward, 2019 http://dyndom.cmp.uea.ac.uk/dyndom/dyndomDownload.jsp)
GraphPad Prism Version 9.0.2 (134) (GraphPad Software Inc http://www.graphpad.com)

Fiji Version 2.0.0-rc-69/1.52n (Schindelin et al., 2012 http://imageJ.nih.gov/ij/)
CRaQ (Bodor et al., 2012 NA)
Image Lab (Bio-rad, https://www.bio-rad.com/de-de/product/image-lab-software?ID=KRE6P5E8Z)
Microcal PEAQ-ITC analysis software
FortéBio Octet BLI Data Analysis software

For manuscripts utilizing custom algorithms or software that are central to the research but not yet described in published literature, software must be made available to editors and reviewers. We strongly encourage code deposition in a community repository (e.g. GitHub). See the Nature Portfolio guidelines for submitting code & software for further information.

Data

Policy information about availability of data

All manuscripts must include a data availability statement. This statement should provide the following information, where applicable:

- Accession codes, unique identifiers, or web links for publicly available datasets
- A description of any restrictions on data availability
- For clinical datasets or third party data, please ensure that the statement adheres to our policy

The cryo-EM map and atomic coordinates have been deposited in the EMDB under accession code EMD-18179 and the PDB under accession code 8Q5H, respectively. Validation report and processed files were provided with the manuscript for peer review.

Research involving human participants, their data, or biological material

Policy information about studies with human participants or human data. See also policy information about sex, gender (identity/presentation), and sexual orientation and race, ethnicity and racism.

Reporting on sex and gender	Not applicable
Reporting on race, ethnicity, or other socially relevant groupings	Not applicable
Population characteristics	Not applicable
Recruitment	Not applicable
Ethics oversight	Not applicable

Note that full information on the approval of the study protocol must also be provided in the manuscript.

Field-specific reporting

Please select the one below that is the best fit for your research. If you are not sure, read the appropriate sections before making your selection.

☒ Life sciences ☐ Behavioural & social sciences ☐ Ecological, evolutionary & environmental sciences

For a reference copy of the document with all sections, see nature.com/documents/nr-reporting-summary-flat.pdf

Life sciences study design

All studies must disclose on these points even when the disclosure is negative.

Sample size	The relevant figure legends report the number of kinetochores for each condition in which fluorescence values were collected. We did not predetermine sample size, nor we calculated sample size. The choice of sample size was based on previous examples in the field, and are consistent with extensive previous experimentation determining epistatic relationships within kinetochores.
Data exclusions	No data were excluded from the analysis
Replication	We indicate the number of technical replicates of each experiment in the relevant figure legends
Randomization	For each immuno-fluorescence analysis, cells (in the indicated number) were chosen randomly for each quantification.
Blinding	The investigators were not blinded during data collection. The same investigators carried out the data collection and data analysis processes.

Reporting for specific materials, systems and methods

We require information from authors about some types of materials, experimental systems and methods used in many studies. Here, indicate whether each material, system or method listed is relevant to your study. If you are not sure if a list item applies to your research, read the appropriate section before selecting a response.

Materials & experimental systems

n/a	Involved in the study
☐	☒ Antibodies
☐	☒ Eukaryotic cell lines
☒	☐ Palaeontology and archaeology
☒	☐ Animals and other organisms
☒	☐ Clinical data
☒	☐ Dual use research of concern
☒	☐ Plants

Methods

n/a	Involved in the study
☒	☐ ChIP-seq
☒	☐ Flow cytometry
☒	☐ MRI-based neuroimaging

Antibodies

Antibodies used

anti-HsNSL1 [mouse monoclonal, clone QL24-1, generated in-house, 1:800]
anti-HsCENP-C (guinea pig polyclonal, MBL-PD030, MBL, 1:1000)
anti-HsNDC80 (mouse monoclonal, clone 93G, ab3613, Abcam, 1:3000)
anti-CENP-T (rabbit polyclonal, SI0822, generated in-house, 1:1000)
anti-HsKNL1 (rabbit polyclonal, SI0788, generated in-house 1:500)
anti-GFP (rabbit polyclonal, generated in house, 1:1000)
anti-Vinculin (mouse monoclonal, clone hVIN-1, V9131, Sigma-Aldrich, 1:10,000)
anti-guinea pig secondary (goat polyclonal, Alexa Fluor 647, Invitrogen A21450, 1:200)
anti-mouse secondary (goat polyclonal, Alexa Fluor 488-conjugated, Invitrogen A11001, 1:200)
anti-mouse secondary (goat polyclonal, Rhodamine Red-conjugated, Jackson Immuno Research 115-295003, 1:200)
anti-rabbit secondary (donkey polyclonal, Alexa Fluor 488-conjugated, Invitrogen A21206, 1:200)
anti-rabbit secondary (donkey polyclonal, Rhodamine Red-conjugated, Jackson Immuno Research 711-295-152, 1:200)
anti-human secondary (goat polyclonal, Alexa Fluor 647-conjugated, Jackson Immuno Research 109-603-003, 1:200)
anti-rabbit secondary (donkey polyclonal, HRP-conjugated, Amersham NA934, 1:10,000)
anti-mouse secondary (sheep polyclonal, HRP-conjugated, Amersham NXA931, 1:10,000)

Validation

Primary antibodies used for western blotting detected proteins with expected sizes confirming the specific binding to the target proteins. Primary antibodies used for IF recognized a signal that disappeared upon RNAi depletion.

-https://www.abcam.com/products/primary-antibodies/hec1hec-antibody-9g3-ab3613.html
-https://www.mblbio.com/bio/g/dtl/A/?pcd=PD030
-https://www.thermofisher.com/antibody/product/A-21450.html?CID=AFLCA-A-21450)
-https://www.thermofisher.com/antibody/product/Goat-anti-Mouse-IgG-H-L-Cross-Adsorbed-Secondary-Antibody-Polyclonal/A-11001
-https://www.jacksonimmuno.com/catalog/products/115-295-003
-https://www.fishersci.com/shop/products/donkey-anti-rabbit-igg-h-l-highly-cross-adsorbed-secondary-antibody-alexa-fluor-488-invitrogen/A21206
-https://www.jacksonimmuno.com/catalog/products/711-295-152
-https://www.jacksonimmuno.com/catalog/products/109-036-003
-https://www.cytivalifesciences.com/en/us/shop/protein-analysis/blotting-and-detection/blotting-standards-and-reagents/amersham-ecl-hrp-conjugated-antibodies-p-06260
-https://www.cytivalifesciences.com/en/us/shop/protein-analysis/blotting-and-detection/blotting-standards-and-reagents/amersham-ecl-hrp-conjugated-antibodies-p-06260

Eukaryotic cell lines

Policy information about cell lines and Sex and Gender in Research

Cell line source(s)

-Sf9 cells (GibcoTMInvitrogen Corporation, Cat. No. 11496-015)
-HeLa cells expressing mCherry-H2B were a gift of Sara Barozzi (Imaging Facility, IFOM-IEO Campus, Milan, Italy) and were not further authenticated.
-DLD1 Flp-In-T-REx also expressing osTIR1 used in this study were received from the laboratory of Don C. Cleveland and were not further authenticated

Authentication

None of the cell lines used were authenticated. The original commercial source of the HeLa cell line is unknown

Mycoplasma contamination

Cell lines were regularly tested for mycoplasma contamination and the test found to be negative

Commonly misidentified lines
(See ICLAC register)

We did not use any misidentified cell line

Plants

Seed stocks	Not applicable
Novel plant genotypes	Not applicable
Authentication	Not applicable
