# Supplementary figures and images for: Structure of the human KMN complex and implications for regulation of its assembly
Source: Nat Struct Mol Biol. 2024 Mar 8;31(6):861–73. doi: 10.1038/s41594-024-01230-9 (PMC11189300; doi:10.1038/s41594-024-01230-9)

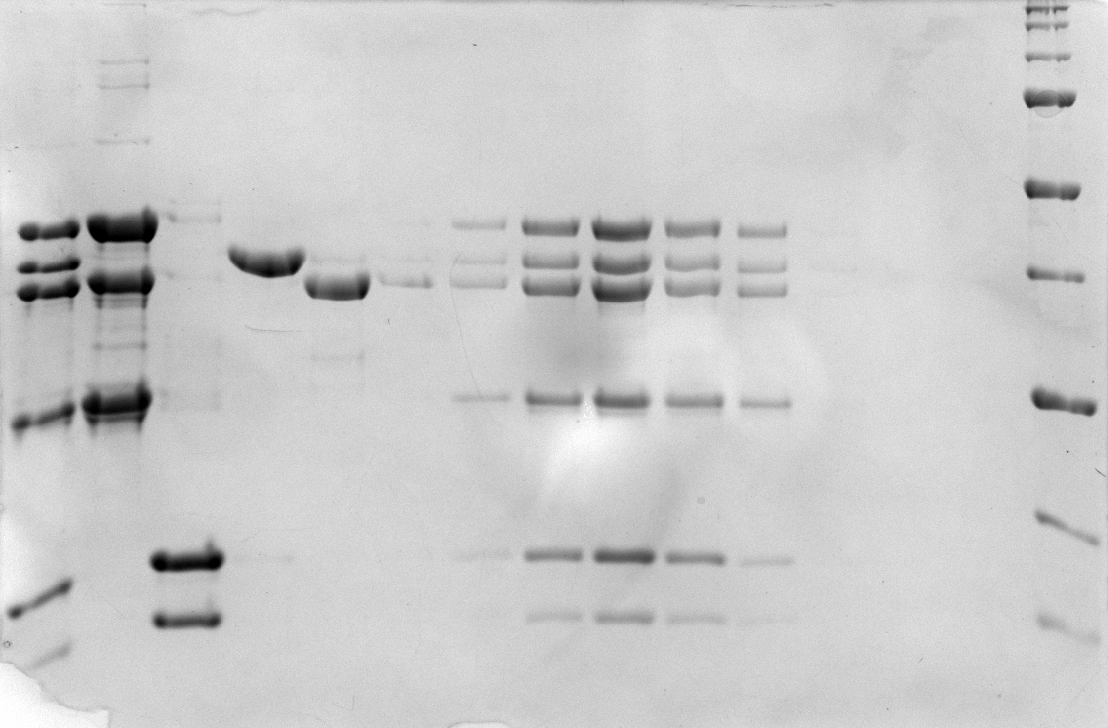

Supplement: Supplementary file 5 — Unprocessed gel. [file 41594_2024_1230_MOESM5_ESM.tif]
