# Supplementary material for: Structure of the human KMN complex and implications for regulation of its assembly
Source: Nat Struct Mol Biol. 2024 Mar 8;31(6):861–73. doi: 10.1038/s41594-024-01230-9 (PMC11189300; doi:10.1038/s41594-024-01230-9)
Supplement: Supplementary file 10 — Unprocessed western blot. [file 41594_2024_1230_MOESM10_ESM.pdf]

Extended Data Fig.4g (chemiluminescence)

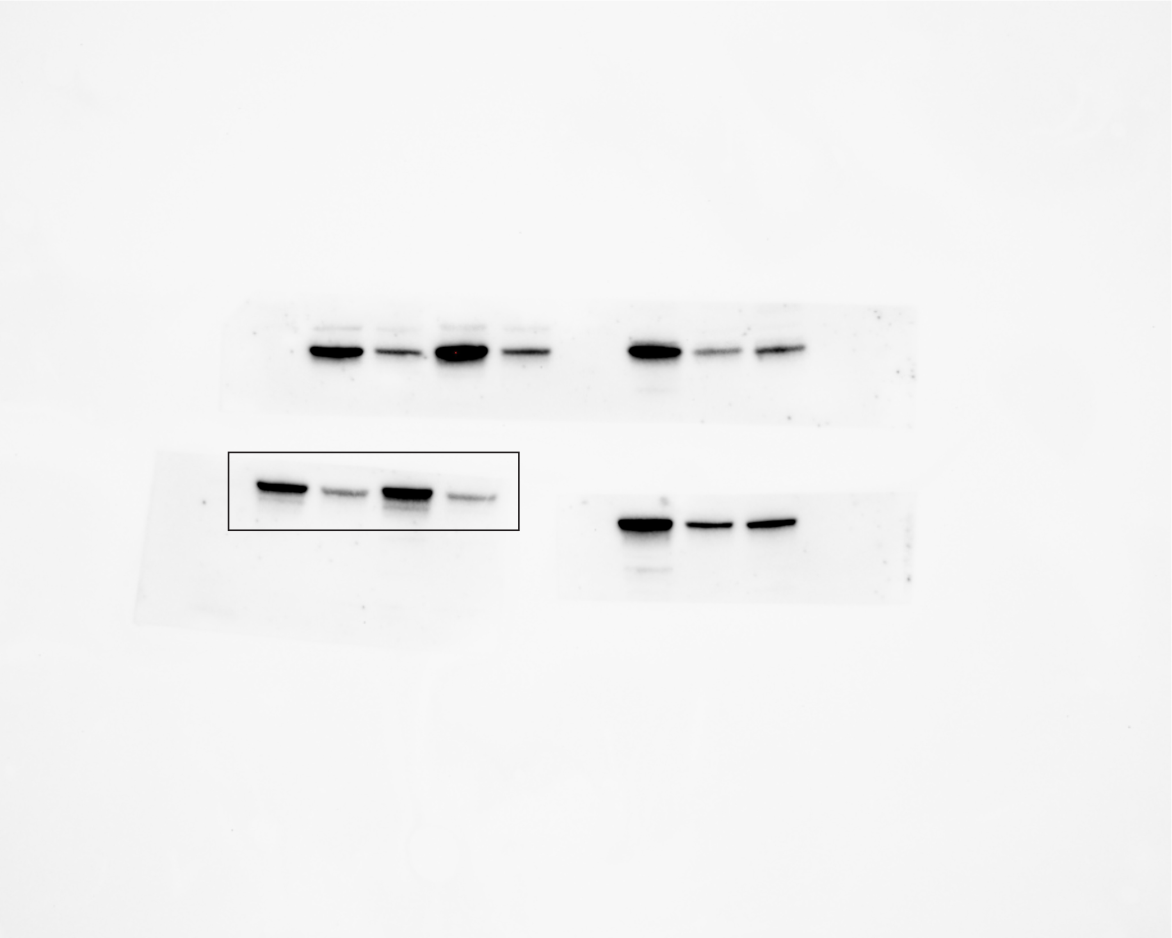

anti-GFP
(1:1000)

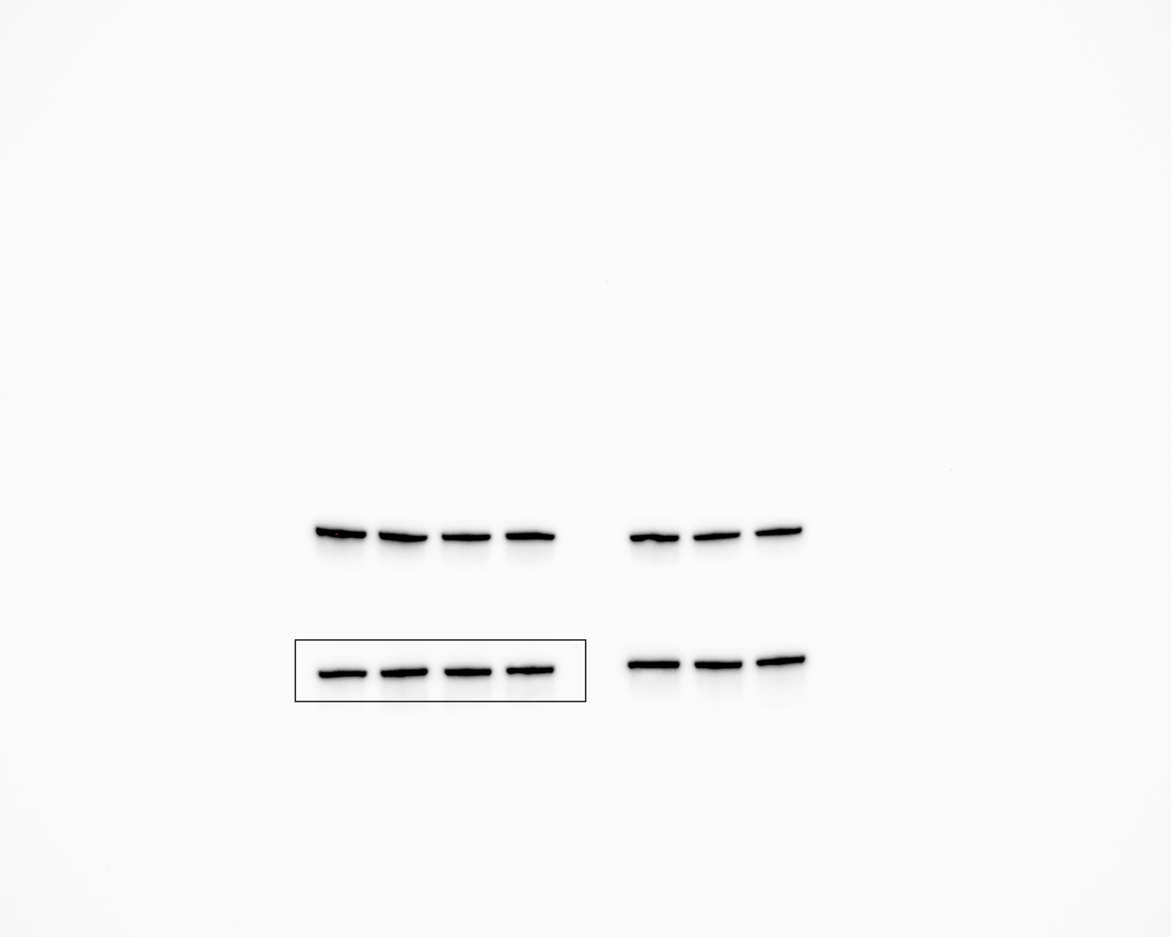

anti-vinculin
(1:10000)

Extended Data Fig.4g (colorimetric)

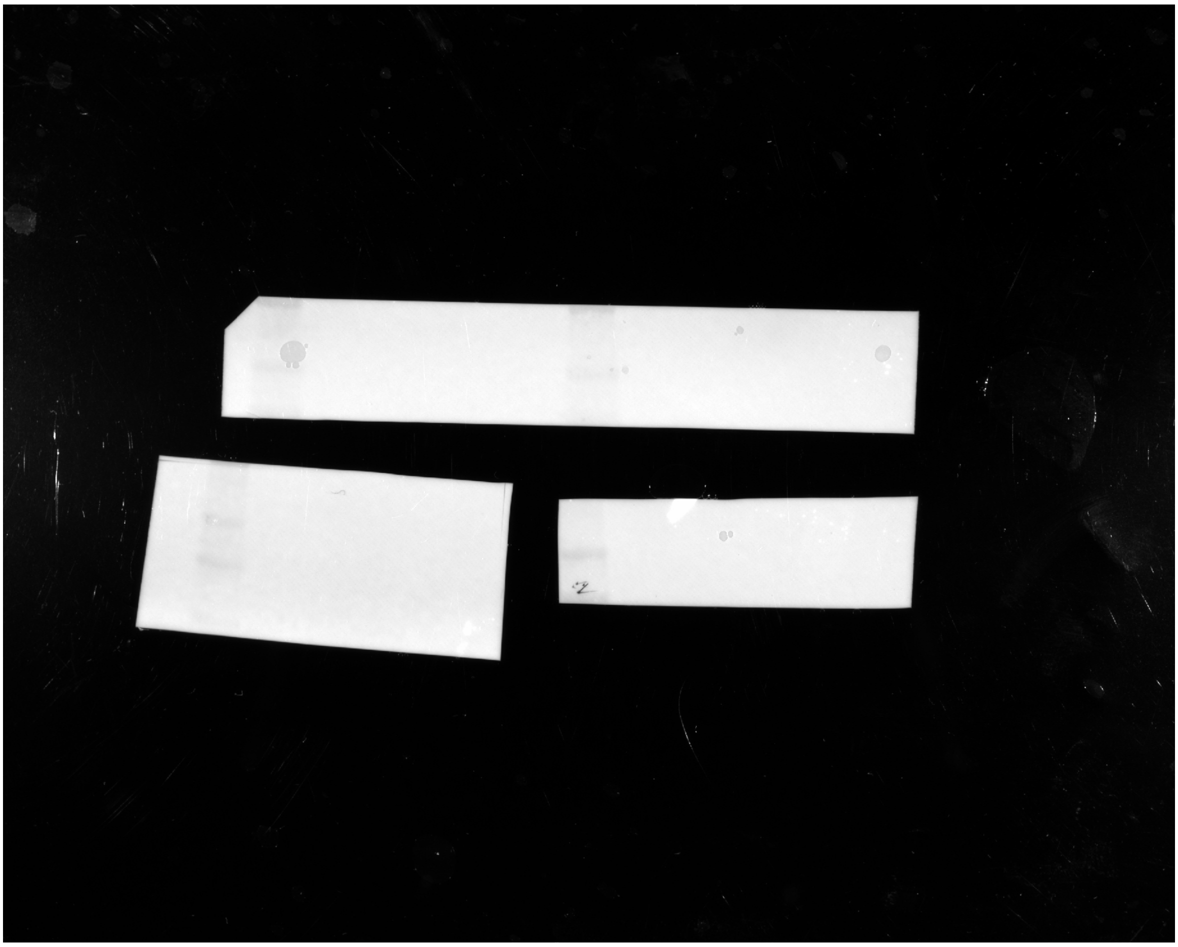

anti-GFP
(1:1000)

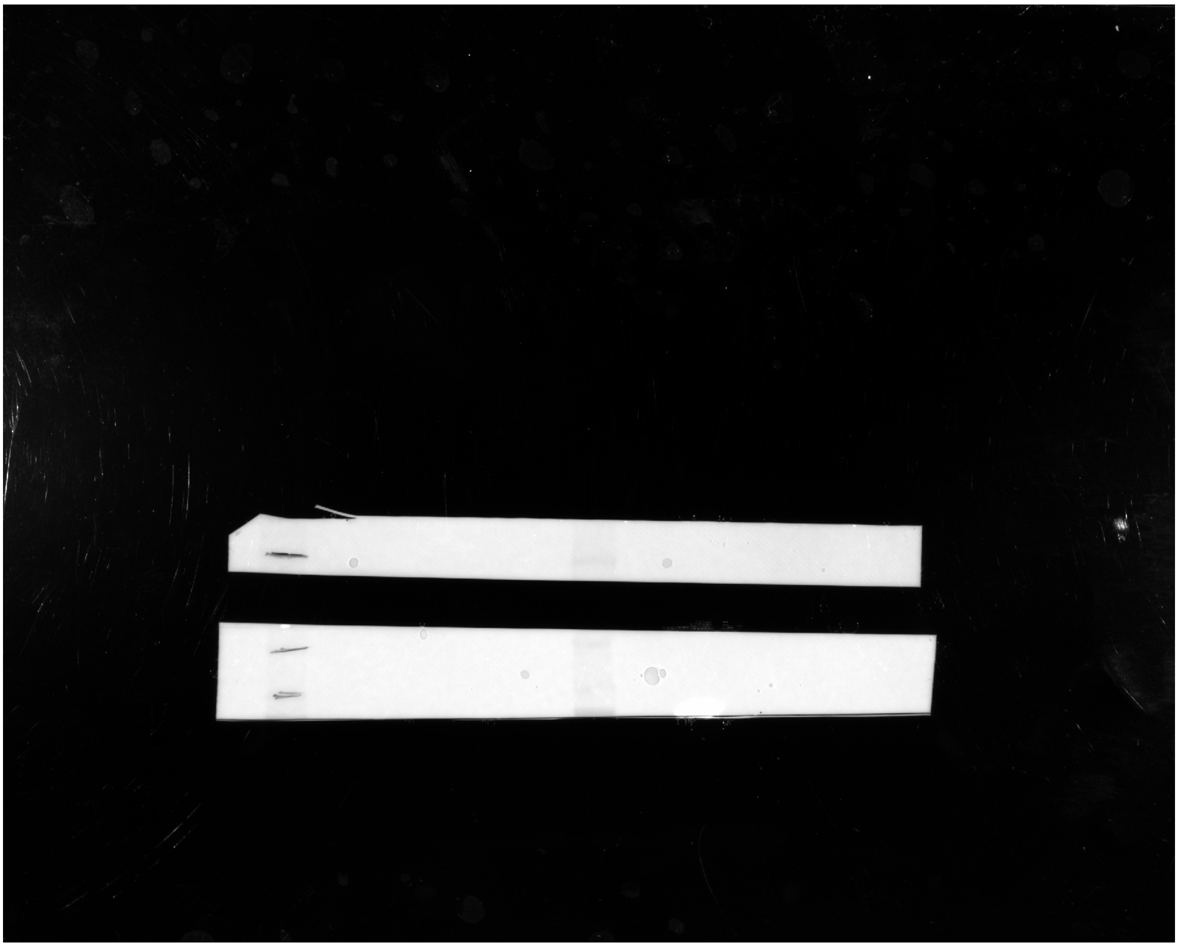

anti-vinculin
(1:10000)

Extended Data Fig.4g (merge)

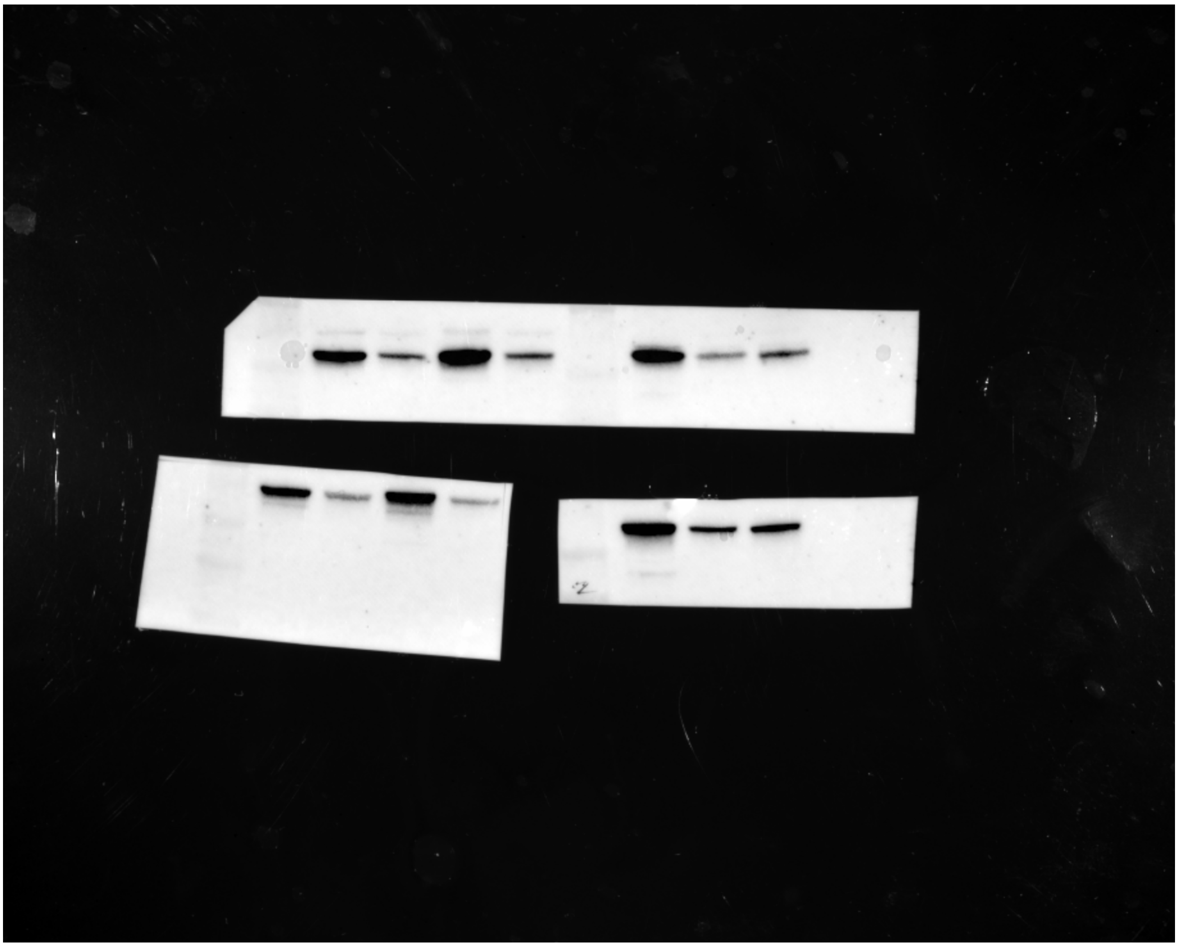

anti-GFP
(1:1000)

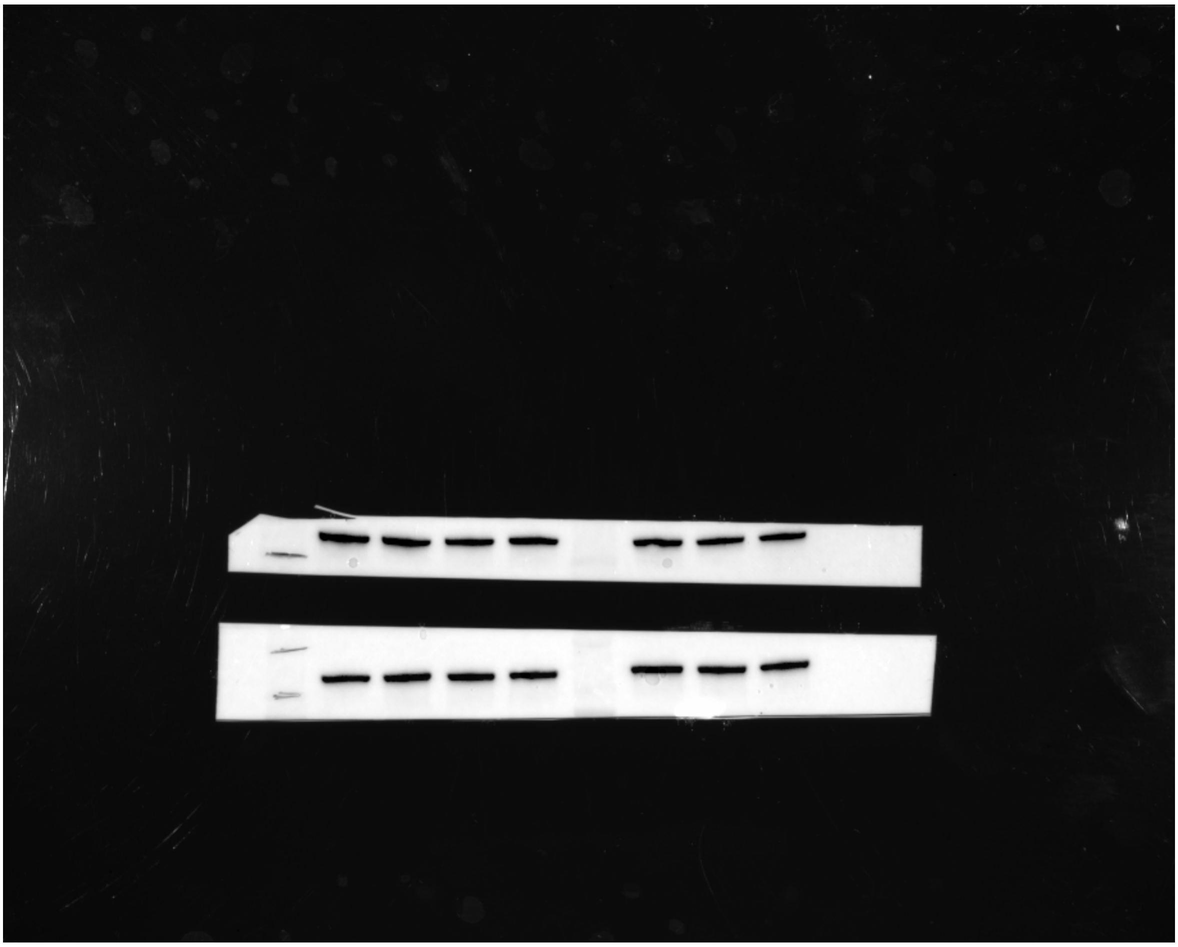

anti-vinculin
(1:10000)
